# Optimization and evaluation of modified release solid dosage forms using artificial neural network

**DOI:** 10.1038/s41598-024-67274-5

**Published:** 2024-07-16

**Authors:** Tulsi Sagar Sheth, Falguni Acharya

**Affiliations:** 1 Department of Applied Sciences and Humanities, Parul Institute of Engineering and Technology, Parul University, Vadodara, Gujarat 391760 India; 2 Parul Institute of Applied Sciences, Parul University, Vadodara, Gujarat 391760 India

**Keywords:** Drug release profile, Similarity factor (f_2_), Artificial neural networks, Simulation, Solid dosage forms, MATLAB, Drug discovery, Medical research, Mathematics and computing

## Abstract

This study aims to optimize and evaluate drug release kinetics of Modified-Release (MR) solid dosage form of Quetiapine Fumarate MR tablets by using the Artificial Neural Networks (ANNs). In training the neural network, the drug contents of Quetiapine Fumarate MR tablet such as Sodium Citrate, Eudragit® L100 55, Eudragit® L30 D55, Lactose Monohydrate, Dicalcium Phosphate (DCP), and Glyceryl Behenate were used as variable input data and Drug Substance Quetiapine Fumarate, Triethyl Citrate, and Magnesium Stearate were used as constant input data for the formulation of the tablet. The in-vitro dissolution profiles of Quetiapine Fumarate MR tablets at ten different time points were used as a target data. Several layers together build the neural network by connecting the input data with the output data via weights, these weights show importance of input nodes. The training process optimises the weights of the drug product excipients to achieve the desired drug release through the simulation process in MATLAB software. The percentage drug release of predicted formulation matched with the manufactured formulation using the similarity factor (f_2_), which evaluates network efficiency. The ANNs have enormous potential for rapidly optimizing pharmaceutical formulations with desirable performance characteristics.

## Introduction

Drug release and dissolution are critical for the dosage forms like tablets, capsules, creams, ointments, and implants^1^. Modified-release (MR) dosage forms are designed to release the drug gradually and steadily over the prescribed duration, and ensures that the medication remains effective over a prolonged period, without causing any adverse effects or sudden spikes in the drug concentration in the body^2,3^. Several process and formulation variables are involved in the drug development process; the best combination of ingredients is found by using the multivariate optimization method^4^. The pharmaceutical industry is concentrating on developing new technologies for oral drugs at low-cost and at the minimal amount of time^5^. Currently, Pharmaceutical formulation development relies on trial-and-error techniques; the discovery, development and maintenance of this process require a lot of time, money, and labour^6,7^. However, with the right approach and resources, it is possible to streamline this process and make it more efficient. Reducing healthcare costs and increasing production of Active Pharmaceutical Ingredients (APIs) can be quite challenging for the pharmaceutical industry. To develop successful strategies for manufacturing the new drug product, the industry must determine the ideal formulations. Traditional methods such as Response Surface Method (RSM), Composite Experimental Design (CED). Shows challenges in the modelling of these interactions^8^. One of the challenges in modelling the drug formulation is understanding the relationships between process variables and unique pharmacological responses^9^. Pharmaceutical experts may develop crucial features of novel medications, such as higher absorption and controlled administration, through the formulation of drug compositions^10^. The drug release kinetics can be compared using the range of mathematical models including zero-order, first-order, Higuchi model, Hixson Crowell, quadratic, and Weibull models.^11–13^.

Sutariya et al.^14^ studied the benefits of ANNs in pharmaceutical research. ANNs ability to predict complex nonlinear interactions and combine experimental and evidence-based data makes them a valuable tool in solving complex problems^15^. ANNs make predictions, detects trends and, draws decisions on information previously stored in the network. Once trained, the network can predict outcomes for the untested data and gives the best possible result. These makes them ideal for dealing with formulation optimization challenges in the development of drug products^16^. ANNs used to optimise drug release characteristics by several authors as reported in the literature^2,4,17–19^.

As a universal approximator, Multilayer Perceptron (MLP) can approximate any nonlinear function with arbitrary accuracy when sufficient processing elements are provided. Prediction of drug release profiles can also be assumed as a function approximation problem and it is achieved by the MLP network. The drug components which meet the desired criteria of drug release can be determined using the Feedforward neural network in the MATLAB software^20^.

This study also compares the drug release characteristics of the predicted formulation of Quetiapine Fumarate MR tablets to the commercially available drugs using a similarity factor (f_2_). The present research highlights the importance of process and formulation variables in determining percentage drug release. This investigation extends the work on Linear Regression Model which also compares the drug release profiles^13^. The structure of the paper is as follows: Section "Preliminaries" presents the preliminaries. Section "Methodology" explains the process of ANNs in drug release kinetics with graphical presentation. In Section "Results" optimum excipient concentration of formulation and process variables on drug release profile is achieved by using MATLAB simulation network. Conclusion is discussed in Section "Conclusion".

## Preliminaries

### Feedforward networks or multilayer preceptor (MLP)

Feedforward networks have one input layer, absent (0) or present (n) hidden layers, and one output layer. In a feedforward network, each neuron in one layer is exclusively directed toward the following layer, referred to as the output layer^21^. Multilayer network architecture is a special case of feedforward neural network. It consists of three layers: an input layer with vector length k, single or many hidden layers with m number of hidden neurons and an output layer with vector length n. For the development of artificial neural networks and the assessment of their accuracy, data was divided into three categories: training, validation, and test data set. The models were built using data from the training, testing, and validation sets, with 70% of the data belonging to the training set, 15% to the testing set, and 15% to the validation set. Data sets for training, testing, and validation were chosen at random by MATLAB software.

**Figure 1:**
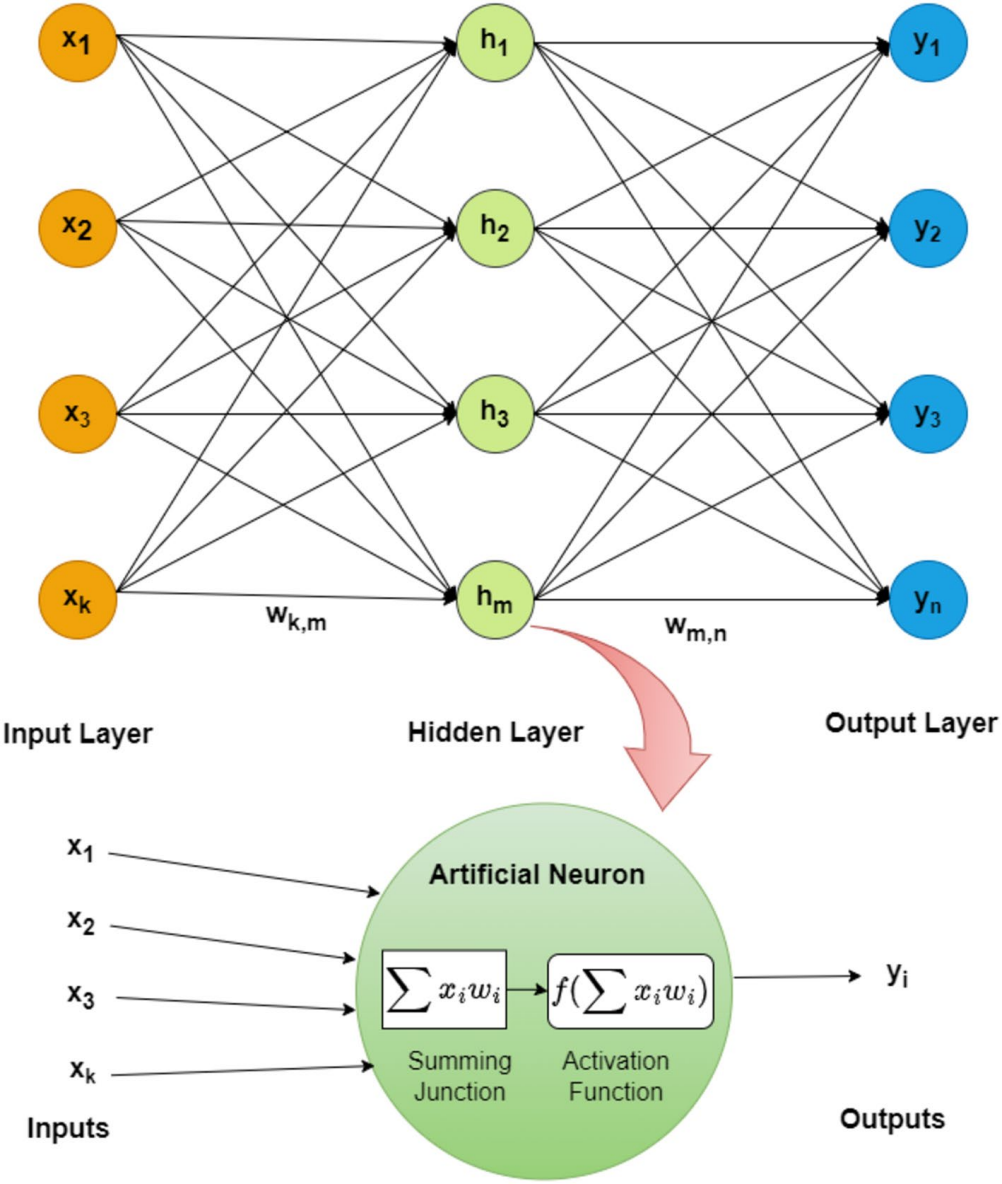
Multilayer neural network architecture and mathematical calculation inside the artificial neuron.

The effectiveness of the created network was evaluated using test datasets. It is essential to note that the test data was not made accessible to the network during training, that is they are the unseen data for the neural network. Figure 1 demonstrates fully connected architecture of a multilayer feedforward neural network with a single hidden layer. The network in Fig. 1 is described as a k-m-n network for simplicity because it consists of k input neurons, single hidden layer with m hidden neurons, and n output neurons.

### Similarity factor (f_2_)

The similarity factor is adopted by USFDA^22,23^ and, is given by Moore and Flanner in 1996^24^. A similarity factor is a logarithmic reciprocal square root transformation of the sum of squared errors. It compares the percentage dissolution of two curves to determine the similarity of two drug profiles^13^.

$${f}_{2}=50 \,log \{{(1+\frac{1}{n}\sum_{j=1}^{n}{({R}_{j}-{T}_{j})}^{2})}^{-0.5} \times 100\}$$

The similarity factor (f_2_) assumes the value 100 when the fit is perfect, and the value decreases when the profiles become more unsimilar. According to the guideline of FDA, the accepted range of f_2_ is between 50 and 100^13,22,23^.

## Methodology

To access the drug release profile, ANN was employed as a part of simultaneous optimization method. The most effective drug release profile for the Quetiapine Fumarate MR tablet was found by analysing the similarity factor f_2_ with that of marketed available drug release profile.

**Figure 2:**
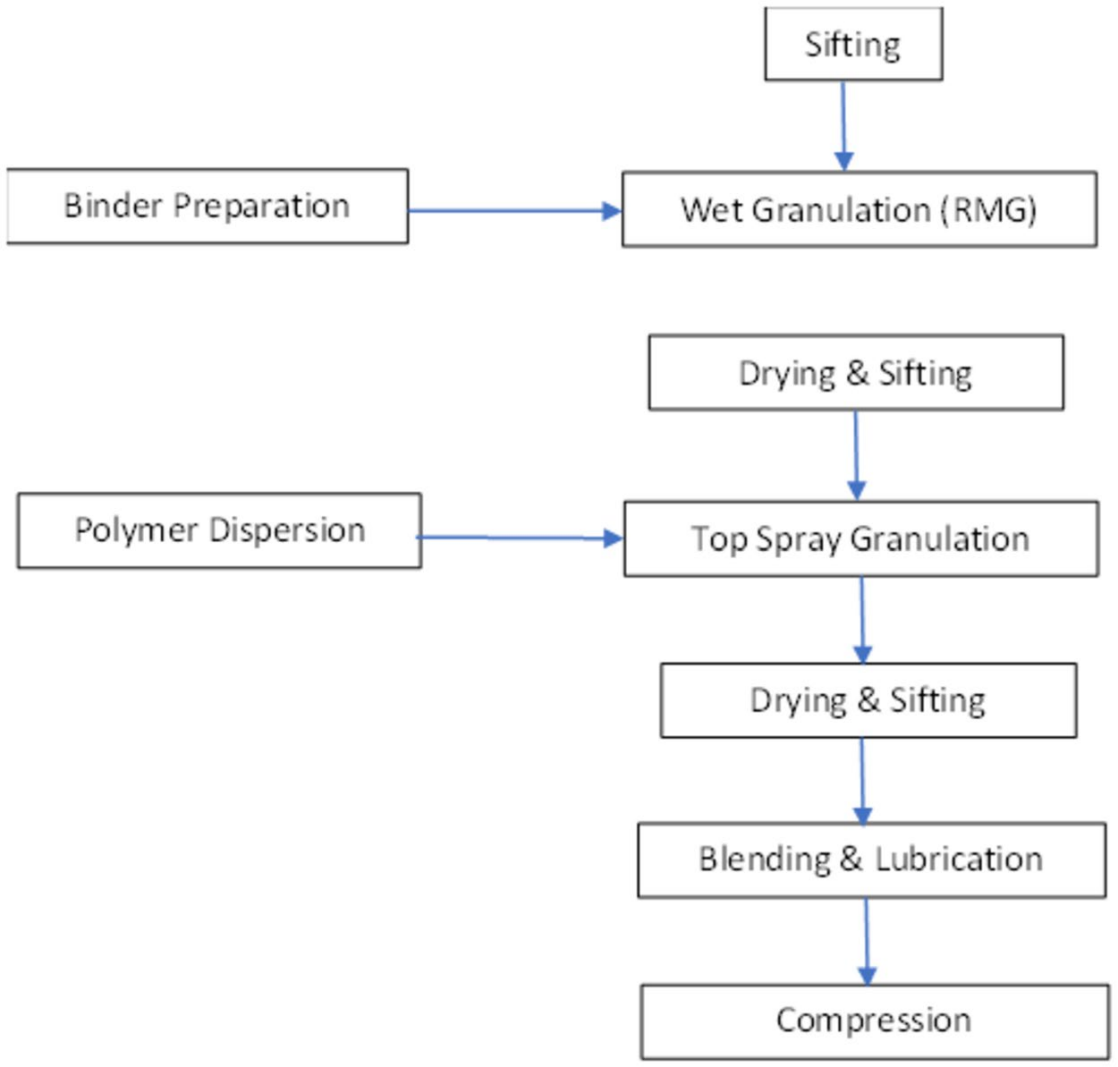
Flow chart of manufacturing process.

The composition of Sodium Citrate (${x}_{1}$), Eudragit® L100 55 (${x}_{2}$), Eudragit® L30 D55 (${x}_{3}$), Lactose Monohydrate (${x}_{4}$), DCP (x_5_) and Glyceryl Behenate (x_6_) was used as a formulation variable. Also, Drug Substance Quetiapine Fumarate, Triethyl Citrate and Magnesium Stearate were kept constant at 230.27, 1.5, and 4 respectively in the training of the network. The concentration of each of the drug content is in mg/tab. The drug content in each formulation is presented in Table 1. The tablet weight kept constant at 400 mg for all the formulations. All possible permutations of the formulation variables within the experimental domain were generated by fractional factorial design. For six variables ${x}_{1}$ to ${x}_{6}$, we get ${2}^{6-2}+1={2}^{4}+1=17$ formulations. All the input variables are simultaneously varied in this approach (See Table 1). The flow chart of manufacturing process is as given below (see Fig. 2).

**Table 1:** Tablet Formulation with different level of Excipient concentration.

Batch no	Sodium citrate ${(x}_{1})$ (mg)	Eudragit® L100 55 ${(x}_{2})$ (mg)	Eudragit® L30 D55 ${(x}_{3})$ (mg)	Lactose Monohydrate ${(x}_{4})$ (mg)	DCP ${(x}_{5})$ (mg)	Glyceryl behenate ${(x}_{6})$ (mg)
F1	**75**	10	15	8.5	15.73	40
F2	50	**5**	15	38.5	15.73	40
F3	50	**15**	15	28.5	15.73	40
F4	50	10	**10**	38.5	15.73	40
F5	50	10	15	53.5	15.73	**20**
F6	50	10	15	13.5	15.73	**60**
F7	25	0	20	40	48.73	**30**
F8	25	0	30	40	38.73	**30**
F9	25	0	30	40	28.73	**40**
F10	50	0	30	40	3.73	**40**
F11	50	0	30	20	3.73	**60**
F12	50	**10**	**10**	38	15.73	40
F13	50	**10**	**20**	28	15.73	40
F14	45	10	15	33.5	**20.73**	**40**
F15	55	10	15	28	**15.73**	**40**
F16	45	10	15	39	**15.73**	**40**
F17	55	10	15	33.5	**10.73**	**40**

Significant values are in bold.

## Results

**Figure 3:**
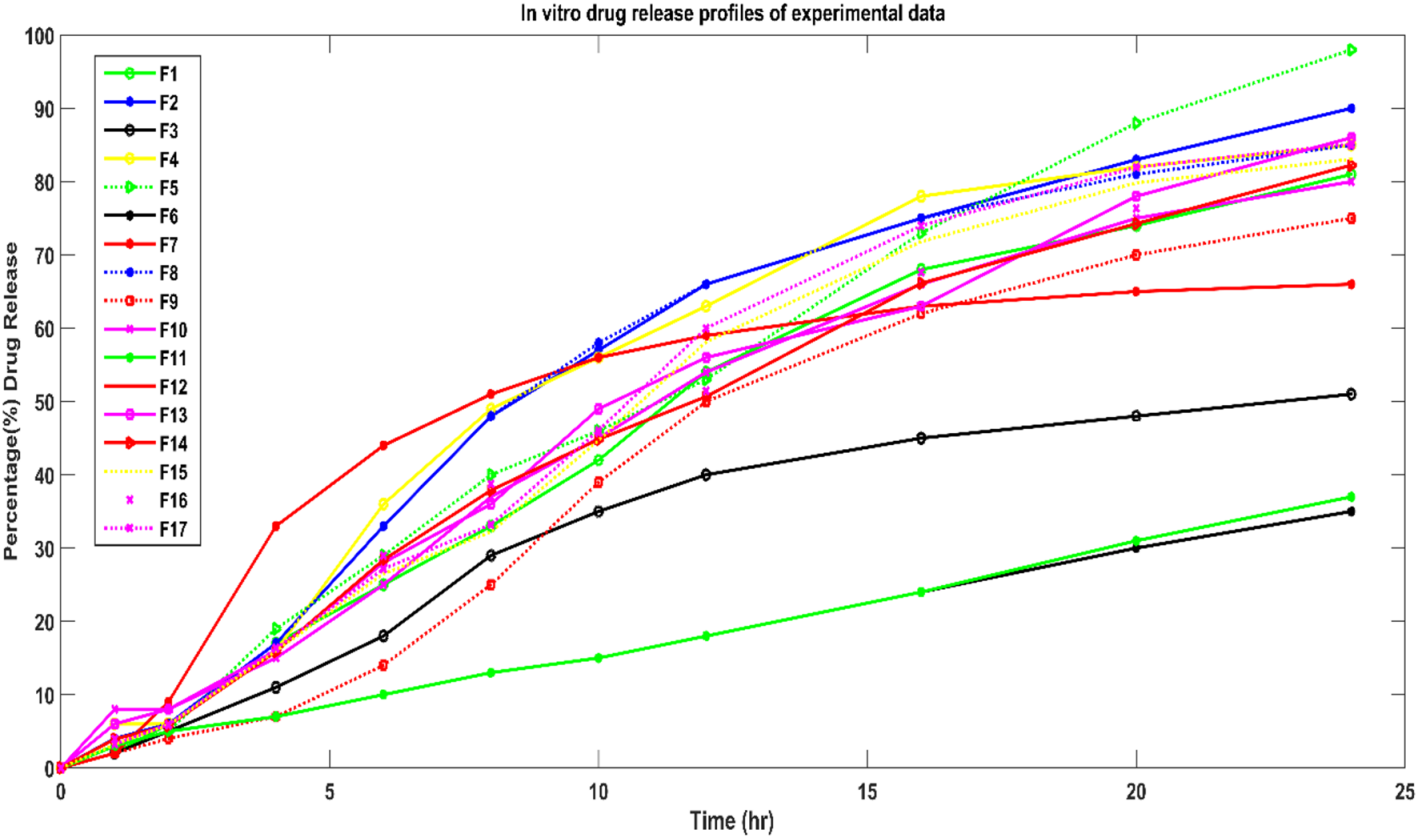
Drug release kinetics for tablet formulation with different level of excipient concentration at ten different timepoints.

**Figure 4:**
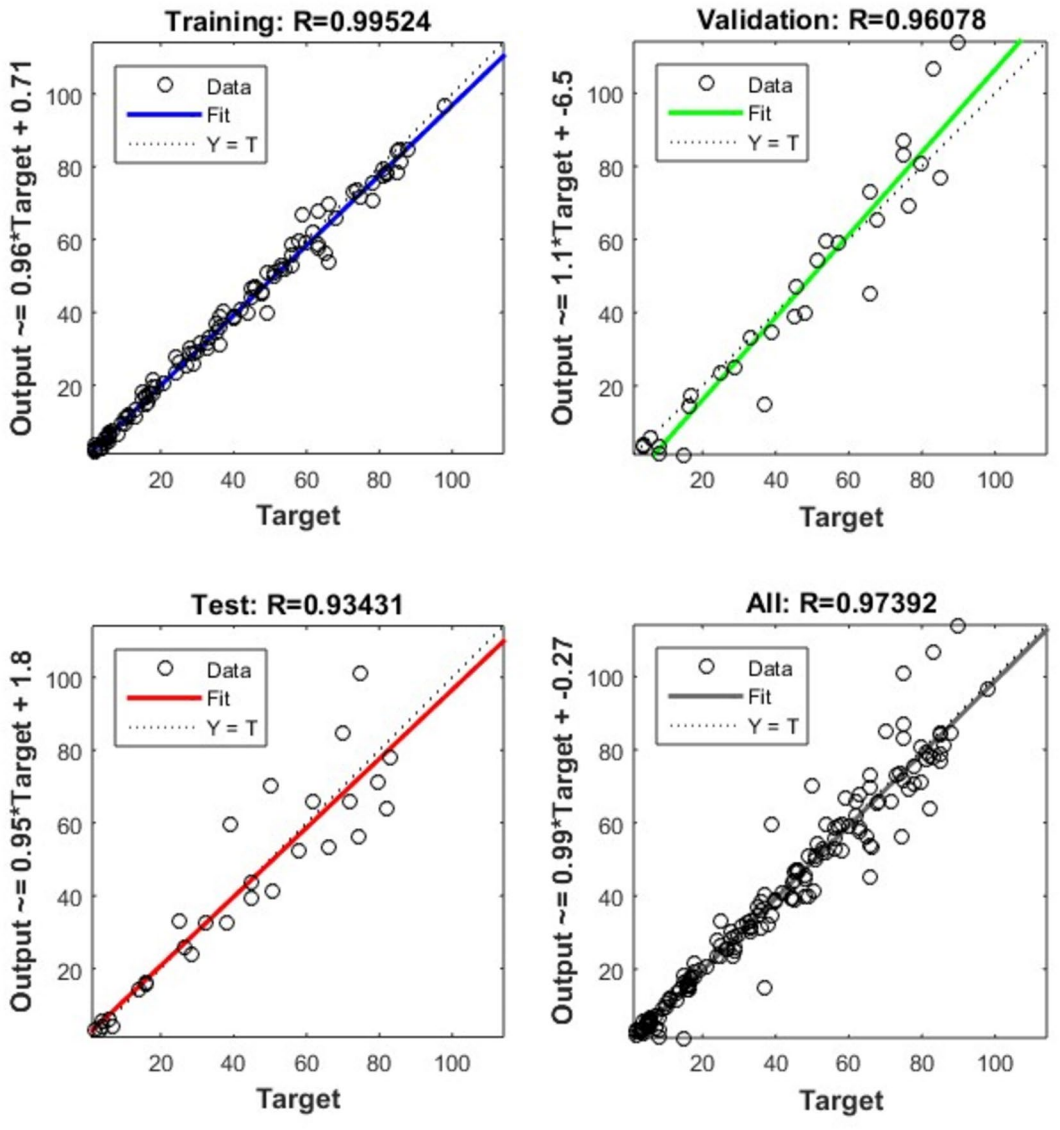
Regression analysis of training, testing and validating data sets with respect to target data.

A formulator can better understand how process variables and formulation variables affect modified-release drug formulation by using conventional (statistical) method but it is time consuming method in creating and developing the modified-release drug formulation. This study found that the ANN model exhibited most suitable approach in checking the drug release similarity by optimizing the formulation variables. The results of the dissolution test for the formulations produced using the fractional factorial design is displayed in Fig. 3. These dissolution profiles were utilized for training, testing, and validating the neural network. The simulation network was processed by MATLAB code^20^. The formulation variable, Sodium Citrate (${x}_{1}$), Eudragit® L100 55 (${x}_{2}$), Eudragit® L30 D55 (${x}_{3}$), Lactose Monohydrate (${x}_{4}$), DCP (x_5_) and Glyceryl Behenate (x_6_) were used as input variables. The drug release profile at ten different time points were used as output. The dimension of input vector and target vector was 6 by 17 (6 variables and 17 samples) and 10 by 17 (10 variables and 17 samples) respectively. “The data set presented in the supplementary table S1 shows the partition of all seventeen formulations in three subsets”. There was total ten neurons in the hidden layer. The Levenberg–Marquardt algorithm was selected to train the network. In MATLAB, the function “trainlm” uses this algorithm for training feedforward neural networks. This algorithm updates weight and bias values during training the neural network. The simulation of trained network (different drug concentration of excipients) was processed when the mean square error is minimum and at this stage the regression coefficient was tending to one for all the trained data sets (see Fig. 4).

The simulated network was used to predict the drug release profiles for the different concentration of drug contents. Also, the change in drug release profile is noted with the different drug concentration of excipients. The percentage drug release is then compared with the drug release of reference drug product. It was noted that, the similarity factor f_2_ is more than 80% for the proposed formulation and, is presented in the Table 2. “The calculation of f_2_ is found in the supplementary table S2 online.”

**Table 2:** Optimized formulation with percentage drug release and similarity factor f_2_.

Optimized formulation		Time (h)	% Drug release	
			Predicted	Reference
Quetiapine Fumarate	230.27	1	3	3
Sodium Citrate	50	2	7	7
Eudragit® L100 55	10	4	18	15
Eudragit® L30 D55	15	6	26	24
Triethyl Citrate	1.5	8	32	33
Lactose Monohydrate	33.5	10	43	43
DCP	15.73	12	55	53
Glyceryl Behenate	40	16	70	68
Magnesium Stearate	4	20	76	78
		24	82	83
		f_2_	85.79	

The steps involved in developing the ANN model is given below:

- Step 1: Data Collection (Experimental data)
- Step 2: Data preprocessing
  - Data cleaning (If any value I not available then put zero)
  - Data normalization
- Step 3: Data Splitting
  - Training (70%)
  - Testing (15%)
  - Validation (15%)
- Step 4: Design ANN model
  - Select ANN architecture
  - Define Input and Output Layers
  - Choose activation functions (TRANSIG, PURELIN)
- Step 5: Training the model
  - Forward propagation
  - Compute loss
  - Backward propagation
  - Update weights
  - Iterate until convergence (train the network until regression coefficient becomes close to 1)
- Step 6: Model validation
  - Validate the model on validation set
  - Tune hyperparameters (number of hidden layers, number of nodes in the hidden layer)
- Step 7: Model evaluation
  - Evaluate model on test set
  - Analyse performance
- Step 8: Optimization
  - Optimize release profile parameters
  - Use trained ANN for prediction
  - Simulate and optimize drug release
- Step 9: Model deployment

## Conclusion

We attempted building an ANN model to predict the impact of formulation excipients on drug release profiles. Various mathematical models are available to check drug release similarity of the dosage form. ANNs were used to find the combination of drug product excipients to predict the percentage drug release. The predicted percentage drug release is compared with the reference drug release using similarity factor (f_2_). Further, the optimal formulation predicted by ANN exhibited the best practice to check influence of drug product excipients on drug release from the tablet. With the recent development, k-nearest neighbor (KNN), Bayesian Algorithm, and Neuro ordinary differential equation can also be applicable in future, as it requires relatively small amount of data and indicates which variables are the most important and the direction that the future experiment should take.

### Supplementary Information

Supplementary Tables.

## Data Availability

The datasets used and/or analysed during the current study are available from the corresponding author on reasonable request.

## Abbreviations

MR: Modified Release
ANNs: Artificial Neural Networks
DCP: Dicalcium Phosphate
APIs: Active Pharmaceutical Ingredients
RSM: Response Surface Method
CED: Composite Experimental Design
MLP: Multilayer Perceptron
FDA: Food and Drug Administration
