# Supplementary material for: Optimization and evaluation of modified release solid dosage forms using artificial neural network
Source: Sci Rep. 2024 Jul 16;14:16358. doi: 10.1038/s41598-024-67274-5 (PMC11252257; doi:10.1038/s41598-024-67274-5)
Supplement: Supplementary file 1 — Supplementary Tables. [file 41598_2024_67274_MOESM1_ESM.docx]

**Optimization and Evaluation of Modified Release Solid Dosage Forms Using Artificial Neural Network**

Tulsi Sagar Sheth^a,b^, Falguni Acharya^c*^

*^ac^Department of Applied Sciences & Humanities, Parul Institute of Engineering and Technology, Parul University, Vadodara 391760, Gujarat, India*

*^b^Research scholar, Parul Institute of Applied Sciences, Parul University, Vadodara 391760, Gujarat, India*

*^a^tulsi.sheth12725@paruluniversity.ac.in,* [*^a^tulsigandhi1@gmail.com*](mailto:atulsigandhi1@gmail.com)*,^c^falguni.acharya@paruluniversity.ac.in*

^*^Corresponding author

**Supplementary Table S1: Tablet formulation with different level of excipient concentration**

| **Batch No.** | **Sodium citrate**  ${(x}_{1})$  **(mg)** | **Eudragit**^®^ **L100 55**  ${(x}_{2})$  **(mg)** | **Eudragit**^®^ **L30 D55**  ${(x}_{3})$  **(mg)** | **Lactose Monohydrate** ${(x}_{4})$  **(mg)** | **DCP**  ${(x}_{5})$  **(mg)** | **Glyceryl behenate**  ${(x}_{6})$  **(mg)** |
| --- | --- | --- | --- | --- | --- | --- |
| **F1** | **75** | 10 | 15 | 8.5 | 15.73 | 40 |
| **F2** | 50 | **5** | 15 | 38.5 | 15.73 | 40 |
| **F3** | 50 | **15** | 15 | 28.5 | 15.73 | 40 |
| **F4** | 50 | 10 | **10** | 38.5 | 15.73 | 40 |
| **F5** | 50 | 10 | 15 | 53.5 | 15.73 | **20** |
| **F6** | 50 | 10 | 15 | 13.5 | 15.73 | **60** |
| **F7** | 25 | 0 | 20 | 40 | 48.73 | **30** |
| **F8** | 25 | 0 | 30 | 40 | 38.73 | **30** |
| **F9** | 25 | 0 | 30 | 40 | 28.73 | **40** |
| **F10** | 50 | 0 | 30 | 40 | 3.73 | **40** |
| **F11** | 50 | 0 | 30 | 20 | 3.73 | **60** |
| **F12** | 50 | **10** | **10** | 38 | 15.73 | 40 |
| **F13** | 50 | **10** | **20** | 28 | 15.73 | 40 |
| **F14** | 45 | 10 | 15 | 33.5 | **20.73** | **40** |
| **F15** | 55 | 10 | 15 | 28 | **15.73** | **40** |
| **F16** | 45 | 10 | 15 | 39 | **15.73** | **40** |
| **F17** | 55 | 10 | 15 | 33.5 | **10.73** | **40** |

**Note:** In Table 1, batch number F1 to F8 and F15 to F17 (Total 11 formulations) were used as input data to train the network; F9, F10, and F14 (3 formulations) were used as testing; F11 to F13 (3 formulations) were used for validation. The above-mentioned data are only an example to give the understanding to the readers. In MATLAB software, these data were selected on the random basis. It is totally blind to the programmer that which data are going for training, testing and, validation; which is a good thing as no one can fix the favourable data in training.

**Supplementary Table S2: Calculation of the Similarity factor f_2_**

|  |  | % Released | |  |  |
| --- | --- | --- | --- | --- | --- |
| n=10 | Time (hr) | Reference | Test | R_j_ - T_j_ | (R_j_ - T_j_)^2^ |
|  | 1 | 3 | 3 | 0 | 0 |
|  | 2 | 7 | 7 | 0 | 0 |
|  | 4 | 18 | 15 | 3 | 9 |
|  | 6 | 26 | 24 | 2 | 4 |
|  | 8 | 32 | 33 | -1 | 1 |
|  | 10 | 43 | 43 | 0 | 0 |
|  | 12 | 55 | 53 | 2 | 4 |
|  | 16 | 70 | 68 | 2 | 4 |
|  | 20 | 76 | 78 | -2 | 4 |
|  | 24 | 82 | 83 | -1 | 1 |
|  |  |  |  | **f_2_** | **85.79** |

**f_2_** =(LOG((1/(SQRT(((SUM(R_j_-T_j_)^2^/n) +1)))*100))*50
